# The Association between Low Levels of Low Density Lipoprotein Cholesterol and Intracerebral Hemorrhage: Cause for Concern?

**DOI:** 10.3390/jcm11030536

**Published:** 2022-01-21

**Authors:** Chen Gurevitz, Eitan Auriel, Avishay Elis, Ran Kornowski

**Affiliations:** 1 Rabin Medical Center, Department of Cardiology, Beilinson Campus, Petah Tikva 4941492, Israel; rkornowski@clalit.org.il; 2 Rabin Medical Center, Department of Neurology, Beilinson Campus, Petah Tikva 4941492, Israel; eitanur@clalit.org.il; 3 The Sackler Faculty of Medicine, Tel Aviv University, Tel Aviv 6997801, Israel; avishayel@clalit.org.il; 4 Rabin Medical Center, Department of Internal Medicine C, Beilinson Campus, Petah Tikva 4941492, Israel

**Keywords:** low-density lipoprotein cholesterol, intracerebral hemorrhage, dyslipidemia

## Abstract

Excessive levels of low-density lipoprotein cholesterol (LDL-C) in the blood are a known risk factor for atherosclerosis, and a common target of treatment for primary and secondary prevention of cerebrocardiovascular disease. As lipid lowering agents including statins, ezetimibe and anti-proprotein convertase subtilisin/kexin type 9 (PCSK9) inhibitors have shown good therapeutic results, the guidelines are constantly lowering the “optimal” LDL-C goals. However, old and new data point towards an association between low LDL-C and total cholesterol and intracerebral hemorrhage (ICH). In this review we aimed to shed light on this troubling association and identify the potential risk factors of such a potential adverse reaction. With respect to the data presented, we concluded that in patients with high risk of ICH, a cautious approach and individualized therapy strategy are advised when considering aggressive LDL reduction.

## 1. Introduction

Intracerebral hemorrhage (ICH), a sub-type of stroke, is a condition whereby a hematoma is formed within the brain parenchyma, and it is associated with a high mortality and disability rate [1,2,3]. Because cholesterol has a key role in the structural formation of cell membranes, low cholesterol concentration has been argued as a potential risk factor for ICH [4]. It has been proposed that low cholesterol results in greater fragility of endothelial cells, which contributes to hematoma extension and slower repair of small intracerebral hemorrhages [4,5]. On the other hand, excessive levels of cholesterol in the blood, and especially its low-density lipoprotein (LDL) fraction, are a known risk factor for atherosclerosis development and augmented rate of cardiovascular events [6]. Thus, lowering LDL cholesterol is a pivotal therapeutic strategy in atherosclerosis management [7]. This review focused on the complex interplay between low levels of LDL cholesterol and the risk of ICH, taking into account the need to lower LDL cholesterol for therapeutic purposes.

## 2. Association from Population Studies

### 2.1. Baseline Cholesterol Level and the Risk of ICH

Over the past two decades, several studies have examined the association between low levels of cholesterol and the risk of ICH. Initially, investigation focused on the association between low levels of baseline cholesterol and its components and ICH. In a pooled prospective study of ICH risk factors published in 2007, with two cohorts combined and over 263,489 person-years of follow-up, LDL cholesterol and triglycerides were moderately inversely related to the incidence of ICH [8].

In the Ibaraki Prefectural Health Study, 30,802 men and 60,417 women, aged 40–79 with no history of stroke or coronary heart disease were assigned and followed for 10 years, and 264 ICH deaths were identified. Participants with LDL-C of 140 mg/dL or higher had half the sex- and age-adjusted risk of death due to ICH compared to those with LDL cholesterol under 80 mg/dL. After adjustment for cardiovascular risk factors, the multivariable hazard ratio compared with people with LDL cholesterol under 80 mg/dL was 0.65 for those with LDL of 80 to 99 mg/dL, 0.48 for 100 to 119 mg/dL, 0.50 for 120 to 139 mg/dL, and 0.45 for 140 mg/dL or higher [9].

A meta-analysis that included 23 prospective studies with a total of 1,430,414 participants and 7960 (5.6%) hemorrhagic strokes, showed a statistically significant inverse association between TC level and risk of hemorrhagic stroke (relative risk (RR), 0.69, 95% confidence interval (CI), 0.59–0.81). An increment of 1 mmol/L in TC concentration was associated with a 15% decreased risk of hemorrhagic stroke. Lower LDL-C concentration was also associated with a higher risk of hemorrhagic stroke (RR 0.62, 95% CI 0.41–0.92). However, no significant association between HDL-C and risk of hemorrhagic stroke was indicated [10].

The relationship between low, usual LDL-C and brain hemorrhage may be further supported by other imaging findings. Cerebral microbleeds (CMBs) are areas of old extravasation of blood detected by T2-weighted gradient-echo magnetic resonance imaging (MRI). Recently, there has been growing interest in this initially considered incidental finding, which is now thought to be associated with an increased risk of ICH. Evidence from the past few years shows that while CMBs are indeed found in around 5% of healthy people, both aging and arterial hypertension are strong risk factors for their presence. Other strong risk factors for CMBs are Alzheimer’s disease, cerebral amyloid angiopathy (CAA), and neuro-inflammation conditions such as multiple sclerosis [11]. A recent Korean study showed that both total cholesterol and LDL-C levels were lower in those with CMBs compared with those without such MRI findings [12].

Other prospective studies have not demonstrated the same association between low LDL-C and ICH or hemorrhagic stroke [13,14,15,16]. The Rotterdam study, for example, in which 9068 stroke-free participants aged ≥55 were followed for up to 20 years, demonstrated that low triglycerides were strongly associated with ICH, and with the presence of deep or infratentorial CMBs on MRI; however, no associations were found for HDL-cholesterol or LDL-C [13].

Moreover, most studies on this topic have been limited by a small number of incident ICH cases. Furthermore, these studies were based on a single baseline LDL-C measurement, which could underestimate the association between LDL-C and ICH risk because of normal fluctuations and changes in LDL-C concentrations over time, particularly due to the initiation of lipid-lowering therapy.

### 2.2. Repeated LDL-C Measurements and the Risk of ICH

Recently, since the emergence of anti-proprotein convertase subtilisin/kexin type 9 (PCSK9) inhibitors, which have revolutionized the field in terms of strategy for lowering LDL-C, the question “HOW LOW CAN YOU GO?” has been raised and re-emphasized again. Consequently, some new studies have been conducted, mostly by collaborations between cardiologists and neurologists. A recent study, which compared two LDL targets (<70 mg/dL and the range of 90–110 mg/dL) in 2860 patients after stroke event, revealed less subsequent cardiovascular events in the lower target LDL group, as expected. As a secondary outcome, the incidence of ICH and newly diagnosed diabetes did not differ significantly between the two groups [17].

Another large prospective study, conducted in China, included 96,043 healthy participants who were free from stroke, myocardial infarction, and cancer at baseline, from 11 centers, with mean age of 51.3 years, and was based on cumulative serum LDL, which was repeatedly assessed every 2 years, for a total of four tests. With a total of 753 identified cases of incident ICH, participants with LDL-C concentration of below 70 mg/dL had a significantly higher risk of developing ICH than those with LDL-C concentrations of 70 to 99 mg/dL; adjusted hazard ratios were 1.65 (95% CI, 1.32–2.05) for LDL-C concentrations of 50–69 mg/dL and 2.69 (95% CI, 2.03–3.57) for LDL-C concentrations <50 mg/dL. The ICH risk was similar for participants with LDL-C concentrations of 70 to 99 mg/dL and those with LDL-C concentrations >100 mg/dL. This association between LDL-C concentration and ICH risk was not significantly modified by age, sex, hypertension status, diastolic blood pressure, BMI, or drinking status, and higher risk persisted consistently for both deep and nondeep hematoma. In an examination of the other lipid profile components, lower (<120 mg/dL) total cholesterol (TC) concentrations were associated with higher ICH risk compared with TC concentrations of 120 to 199 mg/dL, and the associations between HDL-C, triglycerides, and ICH risk were not significant. [18] Indeed, there seems to be an association between low LDL-C and ICH. Of note, most but not all of these data are based on studies conducted in Eastern Asian populations. Nevertheless, this association does not necessarily connect LDL-C lowering with specific agents and ICH incidence.

## 3. Statins and Ezetimibe Clinical Trials and Intra-Cerebral Hemorrhage

Statin clinical trials were conducted largely in patients without prior cerebrovascular disease and changed the field of cardiovascular disease prevention. Phase III trials such as Pravastatin or Atorvastatin Evaluation and Infection Therapy (PROVE-IT), Reversal of Atherosclerosis With Aggressive Lipid Lowering (REVERSAL), A Study to Evaluate the Effect of Rosuvastatin on Intravascular Ultrasound-Derived Coronary Atheroma Burden (ASTEROID), Justification for the Use of Statins in Prevention: An Intervention Trial Evaluating Rosuvastatin (JUPITER), and Myocardial Ischemia Reduction with Aggressive Cholesterol Lowering (MIRACL), showed that lowering LDL-C to <70 mg/dL halted or even reversed the development of atherosclerotic plaque and reduced heart attack and stroke rates. In all of those trials, there was no increase in the incidence of hemorrhagic stroke. [19,20,21,22,23] Furthermore, the first remarkable meta-analysis that explored the association between statin therapy and ICH showed there was no significant increase in ICH among the treated patients [24].

However, while statins cause a dose-dependent reduction of LDL cholesterol, they also have dose-dependent pleiotropic effects, including antithrombotic activity, by inhibiting platelet aggregation and enhancing fibrinolysis and the anticoagulation effect [25,26,27]. Several concerning findings were observed regarding statin use in the last decade. First, an increase in the incidence of hemorrhagic stroke among patients with cerebrovascular disease treated with simvastatin (40 mg) was noted in the Heart Protection Study (HPS), although this was limited by a very small number of events (21 in the simvastatin group versus 11 in the placebo group). [28] Furthermore, a post hoc analysis of the Stroke Prevention by Aggressive Reduction in Cholesterol Levels (SPARCL) clinical trial indicated that atorvastatin use was associated with an increased risk of ICH (but not in fatal ICH) in patients enrolled after experiencing a hemorrhagic stroke, in addition to higher risk of ICH in men and with increasing age. [29] Nonetheless, the small number of patients with brain hemorrhage at entry into this study precludes any meaningful definitive conclusion regarding the relative risks of statin treatment in such a population. Other limitations of this study were its post hoc analysis, high crossover rate in an intention to treat analysis, and the fact that patients with ICH had a reported high blood pressure in their last clinic visit prior to the ICH event. Interestingly, the SPARCL study did not demonstrate relationships between hemorrhage risk and baseline LDL-C or recent LDL-C level in treated patients. Another numerical support (albeit not statistically significant) was found in a few meta-investigations as follows: a large meta-analysis of statin clinical trials [30], a recent meta-analysis of non-statin clinical trials of further lowering LDL-C in patients starting with very low levels [31], and an older Cholesterol Treatment Trialists’ (CTT) Collaboration meta-analysis of more intensive reduction of LDL-C with statin therapy [7], all showing a non-significant positive association between statin treatment or LDL-C lowering and increased risk of hemorrhagic stroke (pooled RR, 1.14 per 1.0-mmol/L LDL-C reduction, 95% CI 0.96–1.36, RR 1.11, 95% CI 0.87–1.43, and RR 1.15, 95% CI, 0.87–1.51, accordingly).

While those meta-analyses showed an insignificant association, others, which focused on high-potency statins, demonstrated significant results; in a small meta-analysis, significant risk of ICH was observed in subjects with high dose of statins (RR = 1.53; 95% CI, 1.16–2.01; *p* = 0.002). [25] The latest meta-analysis on the topic, which examined 33 studies involving 203,305 subjects, indicated that high-dose statin treatment (statin doses that achieved <35% reduction in LDL-C) significantly increased the risk of ICH (RR, 1.35; 95% CI, 1.08–1.68). The analyses, however, did not detect any association between low-dose statin treatment and ICH (RR, 1.05; 95% CI, 0.88–1.25) [32].

Based on these data, a mathematical decision analysis model by Westover et al. suggested that because of the high risk of recurrent ICH in survivors of prior hemorrhagic stroke, even a small amplification of this risk by use of statins suffices to recommend that they should be used with special caution or even be avoided after ICH. For non-traumatic lobar ICH in particular, which is mostly due to CAA (whereas hypertensive vascular disease is primarily responsible for deep ICH), and has a substantially higher recurrence rate than does deep ICH, statin therapy is predicted to increase the baseline annual probability of recurrence from approximately 14% to approximately 22%, offsetting the cardiovascular benefits for both primary and secondary cardiovascular prevention. Overall, life expectancy was calculated higher when avoiding statins in those exceptional circumstances [33].

Another support for this paradigm was demonstrated in a recent large meta-analysis of 39 lipid-lowering trials, which included statins, fibrate, ezetimibe, PCSK9 inhibitors and cholesteryl ester transfer protein (CETP) inhibitors. Whereas lipid lowering therapy was not associated with a statistically significant increased risk of ICH in primary and secondary prevention trials combined, it was associated with an increased risk of ICH in the component of secondary prevention trials (OR, 1.18; 95% CI, 1.00–1.38), although the test for interaction was not significant (*p* 0.31). [34] The association found in the secondary prevention group may also be explained by patients with underlying cerebrovascular disease, with a stroke as their first event. This meta-analysis implies the root for the association discussed lies in merely the lowering of LDL-C, and not in the type of agent used.

Nevertheless, in the only large population-based cohort to date, Saliba et al. showed a protective effect derived from statin use in a dose-dependent manner, using the average atorvastatin equivalent daily dose (AAEDD). [35] While in accordance with previous data, the incidence rate of ICH decreased in a dose-response manner with increasing baseline TC and LDL-C; the incidence rate of ICH also decreased in a dose-response manner with increasing AAEDD. Interestingly, in contrast to baseline TC, the absolute change in TC during follow-up showed opposite results, with HR of 0.98 (0.96–0.99) for each 10 mg decrease in TC level. Those surprising results might be explained by clinical effects of statins other than lipid-lowering: e.g., inhibition of inflammatory processes by attenuation of immunologic mediators, neuroprotection by increased nitric oxide levels mediated by upregulation of endothelial nitric oxide synthase, an anti-oxidative effect mediated by reduced lipoprotein oxidation, and improving cerebral blood flow [35].

In the IMPROVE-IT trial, the addition of ezetimibe to statin treatment lowered LDL cholesterol by approximately 24%. A non-significantly numerically higher risk of hemorrhagic stroke was found with simvastatin–ezetimibe combination than with simvastatin monotherapy (difference of 0.2 percentage points; hazard ratio, 1.38; *p* = 0.11), although once again, the number of hemorrhagic strokes was very low [36].

## 4. Recent Data from PCSK9 Inhibitor Trials

Recently, PCSK9 inhibitor phase III studies, e.g., FOURIER and ODYSSEY randomized trials, reported no significant increase in hemorrhagic stroke among the study population despite profound LDL-C reduction. [37,38] The ODYSSEY OUTCOMES STUDY reported 9 (<0.1%) cases of ICH in the alirocumab group versus 16 (0.2%) cases in the placebo group. [38] In a prespecified secondary analysis of the FOURIER trial, focusing on clinical efficacy and safety of achieving very low LDL-cholesterol concentrations with the PCSK9 inhibitor evolocumab, there was a similar adjusted risk of hemorrhagic stroke (<1%), regardless of the achieved LDL-cholesterol concentration, including in the 2669 patients who achieved an LDL-cholesterol concentration of less than 0·5 mmol/L (19.3 mg/dL) at 4 weeks. [39] Of note, while in the FOURIER trial 19.4% of patients and in the ODYSSEY OUTCOMES 3.2% of patients had a history of previous non-hemorrhagic stroke, patients with a clinical history of known hemorrhagic stroke at any time were excluded from participation in both trials [37,38].

Another recent pooled analysis including two trials of PCSK9 inhibitors showed that the use of PCSK9 had not altered the subsequent risk of ICH. [40] However, these reassuring data derived from the large PCSK9 inhibitor trials should be interpreted with caution because of the relatively short follow-up durations (1.0–6.0 years) and small sample size of hemorrhagic stroke incident cases.

## 5. Pathophysiology and Trying to Establish a Causal Association

The role of LDL-C in the pathophysiology of ICH is not clear. It has been proposed that particularly lower cholesterol results in a weakened endothelium, which more readily leads to arterial fragility, hemorrhage, or slower repair after small hemorrhages [4,5]. Potentially weakened endothelium may be more susceptible to microaneurysms, which are the chief pathological finding of cerebral hemorrhages [5]. Histopathological evidence in humans suggests that lower cholesterol concentrations may increase not only the frailty but also the permeability of brain vessel walls, triggering arterionecrosis, microaneurysm formation and, ultimately, ICH [41,42].

Recently, new genetic studies were conducted in an effort to better establish a causal association between low cholesterol and ICH. A recent, large European study constructed one polygenic risk score (PRS) per lipid trait (total cholesterol, LDL-C, HDL-C, triglycerides) using independent genome-wide significant single nucleotide polymorphisms (SNPs) and estimated the effect of each PRS on ICH risk. They reported an inverse association between the genetic load of risk alleles for total cholesterol and LDL-C and risk of ICH in patients of European ancestry. They also found that genetically instrumented higher total and LDL-C were inversely associated with this same risk. Similar associations were observed for both lobar and non-lobar ICH. These results support a potential causal role of LDL-C in risk of primary, nontraumatic ICH [43].

## 6. Conclusions and Take-Home Message

According to our comprehensive literature review, while the association between low baseline LDL-C and the occurrence of ICH seems to be recognized, the causal association between LDL-C lowering therapeutic strategy and ICH remains uncertain. This is especially in view of the new cholesterol lowering agents that have emerged lately, and the ones that are currently in development, geared towards achieving impressive LDL-cholesterol lowering effects and improved clinical results without manifesting excessive ICH-adverse events. Such a development brings hope to the field of cardiovascular and cerebrovascular morbidity and mortality prevention, but it raises new and old safety concerns. Due to the low hemorrhagic stroke incidence in the different trials and meta-analyses presented so far, the data we have to date are not sufficient in order to determine whether LDL-C lowering indeed causes an increase in the ICH hazard. Thus, there is a need for more data with longer follow-up periods that would enlarge the sample size and maybe provide a clearer adverse association. Meanwhile, we think that it would be advisable to adopt a cautious approach regarding excessive LDL-lowering among patients who are considered at high risk for ICH, according to the data available so far: e.g., patients with previous hemorrhagic strokes, especially very elderly patients, patients with uncontrolled hypertension, or patients with imaging evidence of cerebral amyloid angiopathy using brain MRI. When we treat patients with cardiovascular disease in our clinics, setting the therapeutic LDL-C goals, it might be important to consider individualized risk factors for atherosclerosis and ICH hazards together. Such a holistic patient-centered treatment approach could potentially help improve the safety profile of cholesterol lowering management, although data are still awaited to confirm such a therapeutic strategy.
